# Non-Surgical Treatment of the Accessory Sinuses

**Published:** 1913-10

**Authors:** Richard R. Daly

**Affiliations:** Atlanta, Ga.


### NON-SURGICAL TREATMENT OF THE ACCESSORY SINUSES.

By Richard R. Daly, M. D.,
Atlanta, Ga.

The accessory sinuses are subject to inflammations of a character and degree similar to those of mucous membranes wherever found and in addition they are so secluded that the products and secretions of inflammation cannot well be removed.

Without going far into their anatomy, it is to be noted that they are all larger than the drainage openings leading from them into the nasal cavities and that these openings are hidden away beyond other organs such as the middle turbinates and the unciform processes of the ethmoid. These canals communicate with each other as do the sinuses themselves. The sinuses are also related to the delicate muscular and circulatory arrangements of the eye and to the apparatus for the warming and moistening the air in the nose.

Within the nose, their openings lie chiefly within the "Vicious Circle" that Ballenger describes as located around the anterior end of the middle turbinate where the infundibulum, the openings of frontal, anterior ethmoidal and maxillary sinuses are grouped.

The scroll of the mid turbinate covers most of these and it must be remembered that the turbinates are covered with erectile tissue. Thus we have in our mind a picture that cannot be seen, but every detail of which must be familiar if the diseases of the parts are to be readily comprehended.

The pathology of inflammation within the sinuses is practically the same as that of the mucous membrane anywhere. It is catarrhal or purulent; it greatly modifies function and it may result in the formation of new tissue and the destruction of the old. New growths of both malignant and non-malignant types may appear.

The peculiar feature is that sinuses are comparatively large places connecting with the nares by small openings so that exudates and secretions are likely to dam themselves within the areas and add pressure to the symptom complex.

Within the inflammations producing new growths and extensive granulations, surgery alone is competent to deal when the neoplasms have developed, but before that time and while the early modification of function is chief symptom, even these can be prevented. When one has seen the extensive destruction of tissue and the scars that result from external operations about the nose and brow, or when one has experienced the danger and injury that often accompany operations on the frontal sinus from within the nose, anything that will give relief from these operations is welcome even to the skilled operator. Indeed the more one opens these cavities, the less does he enjoy it.

In acute inflammations of the sinuses, we have pain, some interference with breathing, congestion of all the mucosae within the nose, increase in secretions followed by more or less complete retention of the sinus secretions within the cavities. The general symptoms are those of toxemia. An infection is present. Inspection of the nares shows hyperaemia of all the area of the vicious circle and the indication is to reduce the turgescence and to disinfect the parts. Drainage and antisepsus to assist the phagocytes in fighting off the invasion.

In chronic inflammations where there is continued muco-purulent secretions coming from unseen scources and draining mostly into the epipharynx we have the "catarrh" so dreaded by the patient with its annoyance and debilitation and its general distress. Accompanying this there is likely to be ocular disturbances involving the muscles and the coats of the eye. There may be severe headaches attributable to eye strain, indigestion, toxemia and faculty metabolism.

Within the nose there appears a slight secretion clinging to the midturbinate or seen between it and the septum or between it and the ethmoid cells. The mid turbinate will be hyperplastic or hypertrophic, and all the mucosae will be abnormal in color and reaction. Here again the indication is to reduce the swollen tissues and to wash out the sinuses. And here again do we find obstruction to drainage perfectly evident all over the vicious circle.

These conditions occur at all ages though they come to us more in adult life. It is to be remembered that infection of the sinuses can begin in childhood and chronic inflammations begin at that time which may run their courses to granulations and polypi within the sinuses in later life.

The treatment of these conditions has had one general principle. Establish drainage and maintain it. But how can that be done?

When cocaine came into use, it was hailed as a cure because of its ischemic properties. The reaction from the drug soon showed that it was of no good use, for the tissues were more hyperaemic after its shrinking effects had passed than they were before. In addition there was the habit formation with all its horrors.

The various supra-renal extracts were welcomed and for a time they seemed to be of great value, but the reaction from their use eventually contraindicated them.

Of all the astringents and antiseptics, nitrate of silver in weak solutions gave the best effects and many cases responded entirely to its beneficent influence, but it was too local in its applications. It could not be introduced within the sinuses without puncturing them.

In seeking for an antiseptic that would creep to the sinuses by capillary attraction, I noted that solutions of argyrol remained in the nose for a long time after application, and that often the patient was bothered by the presence of the drug on the handkerchief an hour after treatment. I was about to give up the use of the applications on this account, when it occurred to me that if it were retained, it must be within the accessory sinuses. Up to that time I had been sparing in its use. About a year ago, I began packing the nares with cotton pledgets saturated in 20% solutions of argyrol and leaving them there for 20 minutes or more in all cases where I had any sinus trouble, and at once beneficial results showed themselves.

The turgid turbinates responded very quickly. Immediately after each application they were shrunken and by the following day there was no unpleasant reaction. In acute cases the first treatment would often produce striking results, and relief of pain was immediate, even if there continued to be hypersecretional and other unpleasant symptoms. In chronic conditions, the muco-pus soon disappeared and the dull pains followed in a few days. Where there had been almost constant headache due to unobserved or undiscovered sinusitis, it yielded as soon as the silver salt had opportunity to reduce the swelling within the diseased area.

The method of application is simple. The nares are washed out with a gentle warm douch whenever the secretions are profuse enough to indicate it, but often this is not necessary. Then a filament of cotton is lightly wound on to a slender applicator so that when finished, it looks somewhat like a piece of soft, coarse string with a wire inside it. This is saturated with argyrol 20% and gently tucked between the midturbinate and the septum till one end engages by friction. Then with a little twist the applicator is loosened from the cotton and the long slender piece is gradually tucked into the slit-like passage. The cotton will thus follow the curves of the opening and completely invest the surfaces. If indicated, the cotton is tucked beneath the scroll of the midturbunate in the same manner. The anterior end of the tampon rests in the vicious circle, or if needed another saturated piece can readily be placed there. Of course this all must be done gently. Sometimes there is pain on the mildest application, and ocasionally a weaker solution of the argyrol is required. After it has been present for a few minutes, sneezing may occur, but beyond cautioning the patient not to get any of the medicine on the linen, there is nothing to be done at that time. After 20 or 30 minutes, the pledgets are withdrawn and the patient instructed to blow the nose in cotton or gauze instead of the handkerchief. There is almost invariably relief from the pressure symptoms experienced before the treatment commenced. Later, for an hour or more, and in chronic cases, for the entire day sometimes, the argyrol will continue to appear in the secretions, showing that it has thoroughly invaded the sinuses. In argyrol we have an antiseptic and ischemic that does not irritate and that has no unpleasant reaction, and that will go by capillary attraction into places we can not otherwise reach, except by surgery and the dangerous exploratory probe.

I have had many cases within the general description given above, and I had planned to tabulate them in evidence of the value of this method of treatment. In talking with colleagues, however, I found that the method had already been described.

Dr. J. I. Dowling, of Albany, N. Y., published "Nasal Tamponades" in the Homeopathic Eye, Ear and Throat Journal, June, 1910, and "Relief of Glaucoma Simplex by Means of Intranasal Treatment and Surgery" in the same journal, October, 1910.

He there describes the application of argyrol for the purpose of relieving various inflammations of the eye, such as I hinted at earlier in the paper. In addition, he points out a very important factor which is that the tampons left near the sinuses are stained in a peculiar way and that this staining is due to bacterial action upon the solution. The "Dowling Reaction" is described by Dr. C. E. Terry, of Jacksonville, Fla., in the Transactions of the Florida Medical Association, 1909. In brief, it is that whenever germs are present in the sinuses, and so long as they are present, they will discolor the dark brown pledgets of cotton to a yellow spot. It is further shown that streptococci or staphylococci are present in all these cases till they are cured.

Whether the argyrol has sufficient antiseptic power to kill the germs in the sinuses I am not prepared to say, but I am satisfied as a matter of observation that it will reduce the swelling of the mucosae at the sinus openings, that it will enter the sinuses and thus not only establish, but induce and compel drainage, and that the washing of the sinuses is followed by recovery in the great majority of instances where the treatment is thoroughly applied.

708 Empire Life Bldg., Atlanta, Ga.

